# Educational Attainment of Children Born with Unilateral Cleft Lip and Palate in the United Kingdom

**DOI:** 10.1177/1055665620959989

**Published:** 2020-09-29

**Authors:** Sukhraj S. Grewal, Sirisha Ponduri, Sam D. Leary, Yvonne Wren, John M. D. Thompson, Anthony J. Ireland, Andy R. Ness, Jonathan R. Sandy

**Affiliations:** 1 61139 King’s College London Dental Institute, London, United Kingdom; 2 Queen Alexandra Hospital, Cosham, Portsmouth, Hampshire, United Kingdom; 3 NIHR Bristol Biomedical Research Centre Nutrition Theme, Level 3, 1980 University Hospitals Bristol Education Centre, Bristol, United Kingdom; 4 Speech and Language, Bristol Dental School, 1980 University of Bristol, Bristol, United Kingdom; 5 Department of Obstetrics & Gynaecology, Faculty of Health Science and Medicine, 1415 University of Auckland, Auckland, New Zealand; 6 Orthodontics, Bristol Dental School, 1980 University of Bristol, Bristol, United Kingdom; 7 Epidemiology, NIHR Bristol Biomedical Research Centre Nutrition Theme, Level 3, 1980 University Hospitals Bristol Education Centre, Bristol, United Kingdom

**Keywords:** UCLP, educational attainment, cleft outcomes

## Abstract

**Objective:**

This study evaluated association between functional outcomes in children born with unilateral cleft lip and palate (UCLP) and educational attainment.

**Design:**

Cleft Care UK (CCUK) was a United Kingdom (UK) wide cross-sectional study.

**Setting:**

UK Cleft Teams (data collected from all UK sites providing centralized cleft services).

**Patients, Participants:**

Five-year olds born with nonsyndromic UCLP (n = 268).

**Main Outcome Measure(s):**

National tests for educational attainment Key Stage 1 (KS1) undertaken by children at age 7 were linked to CCUK data to describe differences in educational attainment. Associations between functional outcomes and KS1 results were evaluated using regression analysis. We adjusted for birth month, gender, and an area-based measure of socioeconomic status.

**Results:**

Data were available for 205 children with UCLP. These children scored lower than national average (NA) scores across all subject areas, with a 0.62 lower score observed in the Average Point Score (APS; *P* = .01). There was association between being in a lower category for a cleft related outcomes and poorer KS1 results, with a trend for poorer attainment with higher numbers of poor functional outcomes. Those with 3 or more poor outcomes had a −2.26 (−3.55 to −0.97) lower APS compared to those with 0 to 1 poor outcomes.

**Conclusions:**

Children born with UCLP have poorer educational attainment at age 7 across all subject areas though differences were modest. Children with poor functional outcomes at age 5 had worse educational outcomes age 7. Improvements in functional outcomes could enhance educational outcomes.

## Introduction

Cleft lip and palate (CLP) is a common congenital condition that affects children and their families. Direct effects may include difficulties with eating, speech, and hearing. Indirect effects may include social exclusion, teasing, and bullying (Dixon et al., 2011; Wehby et al., 2014; Dardani et al., 2020). In the United Kingdom (UK), about 12 hundred children are born each year with a cleft (www.crane-database.org.uk) and these children are now treated in a centralized service by multidisciplinary teams (Sandy et al., 2012; Scott et al., 2014). There is good evidence that centralization has improved outcomes although there are aspects of care that still need improvement, such as dental caries (Ness et al., 2018). Further, since care extends into adulthood and beyond, the psychosocial status of patients with cleft is an important part of measuring overall treatment outcomes (Acum et al., 2020).

Studies have consistently shown that cleft is associated with poor educational attainment (Broder et al., 1998; Wehby et al., 2015). Poor educational attainment has wide-ranging and long-lasting adverse effects on mental and physical health outcomes as well as vocational and social standing (Davies et al., 2018). In cleft, there are various explanations for the observed association. These include low intelligence (in some cases linked to syndromic clefts), poor outcomes (such as poor hearing), confounding (by lower socioeconomic status [SES]), or discrimination (such as teacher bias or peer bullying) (Richman, 1978; Jocelyn et al., 1996). If educational attainment in those born with a cleft could be improved through policies and interventions, then quality-of-life expectations are likely to be significantly improved. It is currently not known what the targets of such interventions should be, and whether these targets are modifiable by intervention. There is, however, little evidence that those born with nonsyndromic unilateral cleft lip and palate (UCLP) are genetically predisposed to low educational attainment or intelligence (Dardani et al., 2020).

A number of these explanations are potentially modifiable (eg, by improving functional outcomes or reducing discrimination). Functional outcomes are those which are key measures in clinical audit including speech, hearing, nasolabial appearance, psychological measures, and dental caries. Previous studies have been unable to address the role of possible explanations for poorer educational attainment because of various limitations in the design of the studies. These include small sample sizes (Broder et al., 1998), range of cleft phenotype (Persson et al., 2012), inclusion of syndromic clefts, no measures of functional outcome (Broder et al., 1998; Sandy et al., 2012; Persson et al., 2012), no measures of socioeconomic circumstances (Broder et al., 1998; Persson et al., 2012), no measures of bullying (Broder et al., 1998; Sandy et al., 2012; Persson et al., 2012; Wheby et al., 2014), or use of teacher reported outcomes (Fitzsimons et al., 2018). Given the shortcomings of previous studies, we conducted a linkage study of a detailed UK wide cross-sectional study–Cleft Care UK (CCUK) with national educational records. The primary aim of the original CCUK survey was to establish the impact of centralization of cleft care in the UK (Ness et al., 2018). The primary aim of the study reported here was to link to educational records and describe differences in overall attainment in national samples of children born with UCLP. The secondary aims were to explore if there were differences by gender and subject and to examine whether differences in clinical outcomes could explain some or all of any observed differences in attainment.

## Methods

### Cleft Care UK

Cleft Care UK was a national cross-sectional survey run between 2011 and 2012 to assess whether cleft outcomes had improved as a result of centralization of cleft care within the United Kingdom (Persson et al., 2015). All UK cleft teams participated in this study. The data were collected from 19 sites which included all UK cleft activity. This was organized at the time of the study into 11 centralized services with 17 primary operative sites (Scott et al., 2014). Parents provided written informed consent and children gave their assent. The consent included agreement to link to educational records. Ethical approval was obtained (REC reference number: 10/H0107/33, South West 5 REC) and principles outlined in the Declaration of Helsinki were followed. Data were collected at audit clinics from children born with nonsyndromic, complete UCLP using a standardized protocol. Inclusion criteria comprised:

- Five-year-old children with a nonsyndromic complete UCLP including any with soft tissue Simonart’s bands of less than 5 mm.
- Children born between April 01, 2005, and March 31, 2007.
- The children were aged between 5.3 and 5.9 years. The ages were limited to ensure this was comparable to the previous UK wide survey (Sandy et al., 2001; Persson et al., 2015). If a child failed to attend the initial audit clinic, they were invited to attend a subsequent clinic up until the age of 6.5 years.

Exclusion criteria included:

- Associated developmental delay affecting cooperation with procedures.
- Refusal by caregiver to participate in the study.

### Socioeconomic Status

Postcodes were collected that allowed an area-based measure of SES for CCUK participants (UK Government, 2015). This area-based index is derived from measures of income, education, crime, and barriers to housing. It assigns a numerical score from 0 to 100 to each area of the country with a score of 0 representing the least deprived and 100 represents most deprived area.

### Functional Outcomes Measured in CCUK

Eight important functional outcomes were measured in the CCUK study including audiology, speech, dental health, psychological status, and health and lifestyle questionnaires (Persson et al., 2015). The measures are accepted and widely used clinically with previous assessment of reliability and face validity. All the questionnaires used in the study can be sourced from:http://www.bristol.ac.uk/dental/research/lepoh/ccuk/study_materials/.

#### Dentoalveolar relations

The 5-year-old index (Atack et al., 1997) was used to assess the effects of surgery on growth and facial appearance. It focuses on dental-alveolar relationships, using 5 categories from very poor to excellent, collapsed into 3 categories (excellent/good, fair, poor/very poor) for analysis. Study models were assessed by 2 assessors and a composite score derived (Al-Ghatam et al., 2015).

#### Nasolabial appearance

Frontal and profile photographs were used to assess naso-labial appearance. Facial photographs were assessed with a 5-point ordinal scale using the Birmingham institute of paediatric plastic surgery tool and divided into 3 categories (excellent/good, fair, poor/very poor) (Al-Ghatam et al., 2015).

#### Oral health

A standardized oral health questionnaire, based on the original Clinical Standards Advisory Group survey (CSAG) data collection sheet (Sandy et al., 2001), was used to record the number of decayed missing filled teeth (dmft), collapsed into 0, 1 to 3, and 4+. The dmft was used to assess the presence and severity of dental caries in an individual. All observers were consultants in pediatric dentistry who had attended calibration training prior to data collection (Smallridge et al., 2015).

#### Audiology

Pure tone audiometry was used to determine the degree, type, and configuration of any hearing loss by assessing hearing thresholds and results for the best ear recorded; these were collapsed into normal hearing versus any hearing loss.

#### Speech

The Cleft Audit Protocol for Speech-Augmented (CAPS-A) was used by 2 speech and language therapists to assess speech from audio-video recordings. A measure of speech intelligibility/distinctiveness was derived from CAPS-A which was recorded as 0 to 4 but collapsed into 0 (normal), 1 to 2 (different but intelligible), and 3 to 4 (just intelligible or less) for analysis. The CAPS-A also gave a structural score, which provides information on velopharyngeal function and presence of fistulae, and an articulation score, both recorded as 0 to 3, but collapsed into 0 to 1 and 2 to 3 for analysis; these variables were used for sensitivity analysis (Sell et al., 2015).

#### Psychological variables

Questionnaire based Likert scales were used to measure 3 outcomes relating to psychological status, specifically parents perceived low self-confidence of their child, the parent’s perception of their child being teased or bullied, and parental satisfaction with the whole appearance of their child (Persson et al., 2015; Waylen et al., 2015; Waylen et al., 2017).

### Educational Outcomes in Children

In England, there is a national curriculum (UK Government, 2016) determined by the government for both primary and secondary education, which is divided into key stages. The curriculum is divided into Key Stages (KS), with 4 stages occurring between ages 5 to 16 years. Progress is assessed using a combination of teacher-based assessments and national tests. Key Stage 1 (KS1) tests take place in year 2 when a child is aged 6 to 7. Assessments occur across 5 subject areas of writing, reading, speaking and listening, science, and mathematics.

Since 2002, the Department for Education and Skills (DfES) has compiled a National Pupil Database (NPD) using the KS test results of children undergoing state funded education in England. This longitudinal database holds information on individual identifiable children including that at KS1 and can be used to monitor attainment and progression (Florian et al., 2004). Following approval by the DfES Data Management Advisory Panel, linkage of the CCUK sample (born in England) to the KS1 education data on the NPD was carried out using a password protected spreadsheet.

Key Stage 1 Standard Assessment Test scores (SATs) range from levels 1 to 4, with level 2 being the expected level at the end of KS1. Level 2 for reading, writing, and mathematics is further subdivided into 3 parts (A, B, and C) where A is the highest score and C is the lowest with B being the average level of attainment. The primary outcome measure used for comparison in this study was the average point score (APS), which is a measure used to summarize the overall attainment of a child in English (reading and writing), mathematics, and science at each key stage, by assigning each level a numerical score as in Table 1. Secondary outcomes included percentage of pupils achieving level 2 and above and level 2B and above.

**Table 1.** Key Stage 1 (KS1) Levels and Corresponding Point Scores.^a^

Level	Point score
W	3
1	9
2C	13
2B or 2	15
2A	17
3	21
4	27

Abbreviations: W, Working towards level 1; 1, below the level expected at KS1; 2, the expected level for KS1; 3&4, above the average level expected; level 2 is further subcategorized into: 2C = below average, 2B = average level of attainment, 2A = exceeding the level expected.

^a^ The point scores of each child for writing, reading, science, and mathematics are summed and divided by 4 to attain the Average Point Score (APS).

### Statistical Analysis

The data were analysed using Stata Version 15.0 (StataCorp). The CCUK sample was described using frequencies and percentages for gender.

The primary comparison was between the APS for CCUK children and the national averages (NA), using means, standard deviations (SD), and the *P* values obtained from 2 sample *t* tests. The secondary comparison was the percentage of pupils achieving level 2 or above, and level 2B or above, for all the CCUK children using a 2-sample test of proportions.

The association between functional outcomes and APS was assessed using linear regression, with APS treated as a continuous variable. For speech, intelligibility was used for the main analysis, and structural score and articulation for sensitivity analyses. The results are presented as regression coefficients, 95% confidence intervals (95% CI) and *P* values. Two statistical models were used, with model 1 adjusting for month of birth and gender and model 2 adjusting for month of birth, gender, and the SES. The association between the functional outcomes and secondary percentages of pupils achieving level 2 or above was carried out using logistic regression. The same 2 models were fitted in this analysis. In addition, a variable which summed binary versions of the 6 functional outcomes (dentoalveolar, naso-labial appearance, dmft, audiology, intelligibility, and at least 1 of the 3 psychological variables) was created. This represented the minimum number of problems experienced so as to be able to include children with missing data on some of the binary variables.

## Results

### Description of CCUK Data

Two hundred and sixty-eight children were enrolled in CCUK. Of these, 210 children were educated in England and thus undertook KS1 exams at age 6 to 7. Key Stage 1 data were obtained for 206 of the 210 children—a linkage rate of 98%. One child was absent from all the school tests and was therefore excluded from all analyses leaving a total of 205 children included in this study. Two-thirds of the sample (135) were males and all the children included in the study were from the 9 cleft teams within England.

### Comparison of CCUK Children SATs Results With National Data at KS1

Table 2 illustrates the overall differences in the APS between CCUK children and the English NA. There is statistical evidence of differences between the 2 groups. However, the difference in mean APS between the CCUK children and the NA is modest (16.00-15.38 = 0.62). To put this into context, a difference of a sublevel in SATs (2B vs 2A) requires a difference in APS of 2 points so the difference observed equates to a third of a sublevel. Further, girls with cleft and boys with and without cleft had similar APS values. Girls without cleft had a score that was 0.75 higher than boys without cleft.

**Table 2.** Comparison of the Overall Average Point Score (APS) Between the 2 Groups, Cleft Care UK (CCUK) and the National Average (NA), and Further Subcomparison by Gender.

	All			Boys			Girls		
	CCUK	NA	*P* value	CCUK	NA	*P* value	CCUK	NA	*P* value
Mean	15.38	16.00	.01	15.35	15.50	.6	15.43	16.25	.03
SD	3.42	3.46		3.54	3.65		3.18	3.21	
N	205	642 194		135	328 456		70	313 738	

Abbreviations: CCUK, Cleft Care UK; NA, national average; SD, standard deviations.

**Figure 1.**
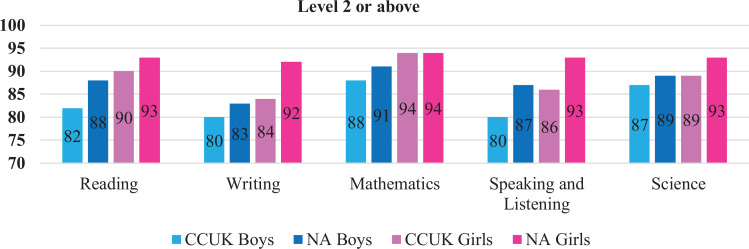
Percentage of pupils scoring level 2 or above in Cleft Care UK (CCUK) versus the 2015 national average (NA) reading: boys *P* = .03, girls *P* = .3; writing: boys *P* = .4, girls *P* = .01; mathematics: boys *P* = .2, girls *P* = 1.00; speaking and listening: boys *P* = .02, girls *P* = .02; science: Boys *P* = .5, girls *P* = .2.

Figure 1 illustrates the differences over the 5 subject areas in achieving level 2 or above. The percentage of pupils achieving level 2 or above is lower in children in CCUK versus NA across all subject areas, and there is statistical evidence to support these differences. Differences in achieving level 2 or above range from 2% to 7% in boys and from 3% to 8% in girls across all subject areas except mathematics and science, where differences are smaller.

Supplemental Figure 2 shows differences between the CCUK children and NA in achieving level 2B and above. Differences vary by gender and subject, with the largest difference being a 14% reduction in attaining level 2B or above in mathematics, among girls. Association between exposure variables and the proportion of pupils achieving level 2 and above in mathematics, speaking and listening, and science are shown in Supplemental Tables 5c, d, and e, respectively.

### Functional Outcomes and Their Association With KS1 Attainment

Table 3 summarizes the key cleft functional outcomes measured in CCUK. These have been divided into categories so that comparison can be made between a change in the category and the educational attainment at KS1.

**Table 3.** Key Cleft Functional Outcomes Measured in CCUK^a^

Variable		Category	Frequency	Percentage
Dentoalveolar, N = 159		Excellent/Good	84	52.8
		Fair	46	28.9
		Poor/Very poor	29	18.2
Nasolabial appearance, N = 193		Excellent/Good	69	35.8
		Fair	105	54.4
		Poor/Very poor	19	9.8
Decayed missing filled teeth (Dmft), N = 203		0	98	48.3
		1-3	61	30.1
		4+	44	21.7
Audiology (best ear), N = 164		Normal hearing	130	79.3
		Any hearing loss	34	20.7
Intelligibility, N = 184		Normal	103	56.0
		Different but intelligible	50	27.2
		Just intelligible or less	31	16.9
Psychological	Parents perceived low self-confidence of child, N = 185	No	170	91.9
		Yes	15	8.1
	Child is bullied, N = 189	No	173	91.5
		Yes	16	8.5
	Parents unhappy with whole appearance of child, N = 188	No	4	2.1
		Yes	184	97.9
Minimum number of above problems, N = 205		0/1	99	48.3
		2	72	35.1
		3-6	34	16.7

^a^ The variable “parents unhappy with whole appearance of child” was not used in subsequent analysis due to very small numbers answering no. The minimum number of problems variables summed binary versions of the 6 functional outcomes (dentoalveolar, nasolabial appearance, decayed, missing, filled teeth [dmft], audiology, intelligibility, and any 1 of the 3 psychological variables).

Table 4 shows the association between functional outcomes and the APS. A negative trend (ie, lower APS for worst outcomes) is seen between cleft functional outcomes and the APS across all of these functional outcomes except bullying. There was statistical evidence for differences in APS by category for each of dmft, intelligibility, and child’s level of confidence that, though attenuated, was still present after adjustment for SES. As the number of poorer cleft-related outcomes increased, so the APS was lower. Those with 3 or more poor outcomes had a −2.67 (95% CI: −3.96 to −1.38) lower APS compared to those with 0 to 1 poor outcomes. This difference attenuated slightly on adjustment for SES to −2.26 (95% CI: −3.55 to −0.97) lower APS compared to those with 0 to 1 poor outcomes.

**Table 4.** Association Between Exposure Variables and the Average Point Score (APS) Assessed Using Linear Regression Coefficients, 95% Confidence Intervals, and *P* Values.^a^

Variable		Category	Model 1 (month of birth & gender)				Model 2 (month of birth, gender, SES)			
			N	Coef	95% CI	*P*	N	Coef	95% CI	*P*
Dentoalveolar (vs excellent/good)		Fair	159	−0.17	−1.34 to 0.99	.8	158	−0.16	−1.30 to 0.97	>.9
		Poor/Very poor		−0.18	−1.57 to 1.20			0.03	−1.31 to 1.37	
Nasolabial appearance (vs excellent/good)		Fair	193	0.04	−0.99 to 1.07	.2	192	−0.04	−1.02 to 0.94	.2
		Poor/Very poor		−1.78	−3.48 to −0.08			−1.64	−3.31 to 0.03	
Decayed missing filled teeth (Dmft) (vs 0)		1-3	203	−1.02	−2.09 to 0.05	.001	200	−0.70	−1.74 to 0.34	.02
		4+		−1.89	−3.08 to −0.71			−1.40	−2.59 to −0.20	
Audiology (best ear) (vs normal hearing)		Any hearing loss	164	−1.01	−2.33 to 0.31	.1	162	−0.90	−2.16 to −0.35	.2
Intelligibility (vs normal)		Different but intelligible	184	−0.45	−1.54 to 0.64	<.001	182	−0.40	−1.45 to 0.65	.001
		Just intelligible or less		−2.97	−4.29 to −1.65			−2.44	−3.72 to −1.16	
Psychological	Low self-confidence	Yes	185	−1.99	−3.80 to −1.72	.03	182	−1.60	−3.36 to 0.16	.08
	Child is bullied	Yes	189	0.09	−1.71 to 1.88	.9	186	0.05	−1.73 to 1.83	>.9
Minimum number of problems (vs 0-1)		2	205	−0.70	−1.70 to 0.29	<.001	202	−0.49	−1.45 to 0.47	.002
		3-6		−2.67	−3.96 to −1.38			−2.26	−3.55 to −0.97	

Abbreviations: Coef., coefficient; SES, socioeconomic status.

^a^ Two statistical models were used, with model 1 adjusting for month of birth and gender and model 2 adjusting for month of birth, gender, and an area-based measure of socioeconomic status (SES).

Tables 5A and B show the associations between the odds of being level 2 or above for reading and writing. The results show a similar pattern to those observed with APS but the associations for reading appear to be stronger than for writing. Those with 3 or more poor outcomes had an odds ratio of 0.20 (95% CI: 0.07-0.53) of achieving level 2 or above in reading compared to those with 0 to 1 poor outcomes. This odds ratio only attenuated slightly on adjustment. Supplemental Tables 5c, 5d, and 5e show the associations between the odds of being level 2 or above for mathematics, speaking and listening, and science. There was no association between number of poor outcomes and performance in mathematics. The associations with speaking and listening were similar to those observed for reading. The association in science was stronger: those with 3 or more poor outcomes had an adjusted odds ratio of 0.24 (95% CI: 0.08-0.77) of achieving level 2 or above in science compared to those with 0 to 1 poor outcomes.

**Table 5A.** Association Between Exposure Variables and the Proportion of Pupils Achieving Level 2 and Above in Reading as Assessed Using Logistic Regression Odds Ratios, 95% Confidence Intervals, and *P* Values.^a^

Reading								
Variable/Category		Max N	Model 1 (month of birth & gender)			Model 2 (month of birth, gender, SES)		
			OR	95% CI	*P*	OR	95% CI	*P*
Dentoalveolar (vs excellent/good)	Fair	159	0.54	0.19-1.52	.4	0.55	0.19-1.56	.5
	Poor/Very poor		0.71	0.20-2.58		0.78	0.21-2.86	
Nasolabial appearance (vs excellent/good)	Fair	193	1.29	0.53-3.12	>.9	1.25	0.51-3.08	>.9
	Poor/Very poor		0.80	0.22-2.91		0.89	0.24-3.32	
Decayed missing filled teeth (Dmft) (vs 0)	1-3	203	0.48	0.18-1.27	.009	0.53	0.20-1.45	.05
	4+		0.28	0.11-0.74		0.36	0.13-1.00	
Audiology (best ear) (vs normal hearing)	Any hearing loss	164	0.38	0.14-0.97	.04	0.38	0.14-1.00	.05
Intelligibility (vs normal)	Different but intelligible	184	0.81	0.25-2.60	.002	0.82	0.25-2.67	.01
	Just intelligible or less		0.17	0.06-0.48		0.20	0.07-0.59	
Psychological	Low self-confidence	185	0.32	0.09-1.09	.07	0.33	0.09-1.15	.08
	Child is bullied	189	1.36	0.28-6.63	.7	1.49	0.28-7.94	.6
Minimum number of problems (vs 0-1)	2	205	0.64	0.24-1.67	.002	0.73	0.27-1.96	.01
	3-6		0.20	0.07-0.53		0.23	0.08-0.65	

Abbreviations: OR, odds ratio; SES, socioeconomic status.

^a^ Model 1 adjusts for month of birth and gender and model 2 also adjusting for an area-based measure of socioeconomic status (SES).

**Table 5B.** Association Between Exposure Variables and the Proportion of Pupils Achieving Level 2 and Above in Writing as Assessed Using Logistic Regression Odds Ratios, 95% Confidence Intervals and *P* Values.^a^

Writing									
Variable/Category		Max N	Model 1 (month of birth & gender)			Model 2 (month of birth, gender, SES)			
			OR	95% CI	*P*	OR	95% CI	*P*
Dentoalveolar (vs excellent/good)	Fair	159	1.00	0.39-2.59	1	1.03	0.39-2.68	.8
	Poor/Very poor		1.03	0.33-3.20		1.13	0.36-3.53	
Nasolabial appearance (vs excellent/good)	Fair	193	0.90	0.38-2.14	.1	0.87	0.36-2.10	.1
	Poor/Very poor		0.31	0.10-1.01		0.34	0.10-1.13	
Decayed missing filled teeth (Dmft) (vs 0)	1-3	203	0.49	0.21-1.19	.02	0.55	0.23-1.34	.08
	4+		0.35	0.14-0.86		0.44	0.17-1.13	
Audiology (best ear) (vs normal hearing)	Any hearing loss	164	0.44	0.18-1.06	.07	0.45	0.18-1.09	.08
Intelligibility (vs normal)	Different but intelligible	184	0.62	0.24-1.58	.03	0.62	0.24-1.61	.09
	Just intelligible or less		0.34	0.12-0.92		0.42	0.15-1.18	
Psychological	Low self-confidence	185	0.61	0.18-2.10	.4	0.65	1.19-2.28	.5
	Child is bullied	189	1.70	0.36-8.08	.5	1.75	0.35-8.73	.5
Minimum number of problems (vs 0-1)	2	205	0.76	0.33-1.75	.04	0.86	0.37-2.02	.1
	3-6		0.36	0.14-0.92		0.43	0.17-1.13	

Abbreviations: OR, odds ratio; SES, socioeconomic status.

^a^ Model 1 adjusts for month of birth gender and model 2 also adjusting an area-based measure of socioeconomic status (SES).

Sensitivity analysis was undertaken using either the structural or articulation score in place of intelligibility. There were 20.5% with structural problems and 29.4% with articulation problems; there were no associations with APS for either variable (regression coefficients [CI]: −0.44 (−1.57 to 0.69) and −0.34 [−1.36 to 0.68], respectively, after adjustment).

A post hoc analysis was undertaken whereby the differences in the APS between the CCUK children who had less than 2 poor functional outcomes were compared with the English NA. The mean (SD) values for all children, boys only, and girls only were 16.08 (3.42), 16.21 (3.41), and 15.88 (3.48), respectively, and there was no statistical evidence for differences when compared with the NA values in Table 2 (*P* > 0.1 for all).

## Discussion

### Summary of Findings

Children born with a nonsyndromic complete UCLP have lower educational attainment overall and across a range of domains at age 7. Differences observed are similar to those reported in previous comparable studies (Wehby et al., 2014). Differences are not large (one-third of a sublevel) which is similar to the gender differences observed between boys and girls (without cleft). Associations were modestly attenuated after adjustment for SES. Children with better functional outcomes had better educational attainment that was similar to that of children without cleft.

### Comparison With Previous Studies

Concerns that children born with nonsyndromic cleft have schooling difficulties were highlighted in a 2-center study in 1998 (Broder et al., 1998). Nearly half of the children had learning disability and poor school achievement and over a quarter had to repeat a grade. This was influenced by cleft type and gender. Males with cleft palate (CP) only and females with CLP were most vulnerable. These findings were supported by a much larger retrospective population-based study in Sweden. This compared academic achievement at the time of high school graduation of 1992 cleft individuals with the general population (1.2 million unaffected children). Cleft type influenced educational achievement and a further analysis of the same data suggested girls were more negatively affected than boys (Persson et al., 2015; Persson et al., 2018). It is difficult to make comparisons across countries and cultures but in most studies, those with CP only have the most negative outcomes, followed by those with CLP and cleft lip (CL) only being the least affected. The influence of gender is variable. Objective educational measures and targets vary from country to country and dissection of the educational issues for those born with cleft requires more detailed studies (Persson et al., 2018).

Wehby et al. (2014) recognized and addressed the need to assess the impact of isolated nonsyndromic clefts on academic achievement using national standardized tests for 588 affected children born with CP, CL, or CLP. Children were matched to unaffected classmates by gender, school/school district, and month and year of birth. Children born with clefts scored lower across all subject areas with a 5% difference in overall composite scores compared to unaffected controls. This is comparable to our findings which included assessment of academic achievement across all subject areas and used an overall composite score. Our study assessed academic achievement at a single key stage 1 (KS1) in children with UCLP, whereas Wehby et al. followed children from grade 2 until the end of high school. A trajectory analysis on these data showed that if children’s academic achievement is tracked from elementary to high school, cleft affected children are more likely to be classified into a persistently low achievement trajectory (Wehby et al., 2014). Predictors of poor academic achievement in studying included less frequent use of prenatal care and a low level of maternal education (Wehby et al., 2015).

Another study showed that children born with isolated clefts and their siblings had similar levels of academic achievement. Furthermore, birth order showed that younger siblings have higher risk of poor academic outcomes (Collett et al., 2014). Findings from these studies suggest that shared socioeconomic circumstances or other shared factors (such as subclinical cleft phenotypes and laterality) explain some of the observed differences in academic achievement (Wehby et al., 2014; Wehby et al., 2015; Persson et al., 2018; Gallagher et al., 2018). Academic achievement of 5-year-old children with isolated clefts in England has been reported by linking the Cleft Registry and Audit Network database with the NPD (Fitzsimons et al., 2018). Children born with a cleft and no additional anomalies in England who were 5 years old between September 1, 2006, and August 31, 2012, had academic achievement which was below the NA for all 6 assessed area. This study included a large sample size (2802) and all types of isolated clefts. However, it assessed children in year 1 and not year 2 which meant that the academic achievement was based on teacher assessments rather than standardized tests. There is therefore potential for bias because it has been known for some time that teachers rate the ability of children born with cleft as lower (Richman, 1978a; Richman, 1978b). Furthermore, the linkage rate of 61% of 5-year was lower than our study but will have included some of our subjects.

### Modifiable Explanations in Educational Attainment

Speech is an important aspect of function and poor speech is linked to social interaction, negative peer reactions, and poorer academic achievement (O’Brian., 2011). Children with cleft have been reported to have poor speech compared to their peers (Knight et al., 2015). Even in a centralized service, around 17% of cleft children had speech that was only just intelligible to strangers or impossible to understand. Differences by subject area are most marked for speech (Figure 1).

Socioeconomic status is an important determinant of educational outcome (Fowler at al., 1985; Resnick et al., 1999; Wehby et al., 2015). Therefore, lower SES may explain some of the associations observed. Though the observed differences could be due to residual confounding by SES, the observation that differences (though attenuated) are still present after adjustment for SES suggest that other modifiable factors may play a role.

Poor oral health has been shown to be associated with negative educational outcomes. A study in North Carolina based on 2871 children showed that children with poor oral health were 2.3 times more likely to report poor school performance (Blumenshine et al., 2008). Our study showed a strong association between a high dmft and poorer educational attainment as shown in Table 4. Possible explanations for these poorer educational outcomes represent confounding by social circumstances or result from poorer concentration at school due to oral pain, missed school days due to caries symptoms or treatment, or discrimination and bullying from other school children. Interestingly, school absence affects school performance for all children and this absence does not differentially disadvantage children born with a cleft (Bell et al., 2017).

Educators also need to be aware of how a child born with cleft may struggle because of emotional and social difficulties as well as the fact they will require hospital appointments in school hours. Teachers need to be informed of the cleft/education literature and may need specific training to support these children (Stock et al., 2019). It is also important to recognize that the views of children born with cleft and their parents as well as health care professionals are not always coincident (Nelson & Kirk, 2013). This is also true in the school setting where children with cleft may view their progress differently to their parents (Stock & Ridley, 2018).

### Strengths of Study

This study has several strengths. First, this was a rigorous study with a good response rate (74%) (Persson et al., 2015). Second, high linkage rate was achieved (98%). Third, a defined single cleft phenotype with no other craniofacial abnormalities was studied thus reducing the chance of low intelligence associated with syndromes confounding our results. Fourth, functional outcomes were measured so that we could assess their explanatory power. Fifth, using the area based measure of SES allowed adjustment for confounding due to SES. Sixth, an objective primary outcome was used, which has been used previously to compare pupils across key stages and has been shown to be a good measure of overall attainment was used (Farrell et al., 2007; Withey et al., 2015). This allowed national comparison and removed potential teacher assessment bias.

### Weaknesses of Study

This study has several weaknesses. First, this study was not large, meaning that some analyses were underpowered. However, for a cleft study which is not based on a registry, this is a large sample with a single phenotype and rich data. Second, multiple comparisons were conducted as secondary analysis that should be interpreted with caution until replicated. Third, missing data further reduced the power and potentially introduced bias. Fourth, only one measure of educational attainment at age 5 was available with no later measures of attainment or career prospects. However, previous research suggests that children continue on a poor academic trajectory (Wehby et al., 2015). Fifth, no measures of intelligence (IQ) or of teacher discrimination were available. Sixth, we did not collect any data on cognitive issues, such as learning disabilities, Attention Deficit Hyperactivity Disorder (ADHD), or language impairment. Finally, the measure of SES has limitations in that it is an area-based measure and therefore may not accurately reflect individual SES.

### Implications of This Study

Interventions to improve functional outcomes in cleft are required in order to improve educational outcomes. Improvements have occurred with centralization of cleft services, but speech is still an issue with around 17% of cleft children having speech that was only just intelligible to strangers or impossible to understand. Other functional outcomes such as hearing and oral health have not improved with centralization. This may reflect the fact that speech and language therapy, audiology, and dental care are not provided centrally suggesting there is a need to develop models of care to ensure an integrated high quality of care is provided.

### Future Research

Larger studies with power to detect important differences in educational attainment are required. Nearly all other studies reporting educational outcomes in children with an oral cleft are based on samples of children with a wide age range (Fitzsimons et al., 2018) which has been mitigated against in the current study. Longitudinal studies could usefully describe tracking and the associations at older ages in order to confirm that children continue in poor academic trajectories. More detailed studies to explore other modifiable explanations (eg, with a measure of IQ) would be valuable. This study assessed a single cleft phenotype, isolated UCLP. Future research could be expanded to include other cleft phenotypes and the influence of cleft laterality on educational attainment.

## Conclusion

Children born with a nonsyndromic complete UCLP in England had poorer educational attainment compared to the noncleft population. The difference observed is modest and potentially modifiable. Future longitudinal studies are needed to further explore the impact of modifiable factors on academic outcomes. Service developments are required to further improve speech, hearing, and oral health in children with cleft.

## Supplemental Material

Supplemental_material - Educational Attainment of Children Born with Unilateral Cleft Lip and Palate in the United Kingdom Click here for additional data file. Supplemental_material for Educational Attainment of Children Born with Unilateral Cleft Lip and Palate in the United Kingdom by Sukhraj S. Grewal, Sirisha Ponduri, Sam D. Leary, Yvonne Wren, John M. D. Thompson, Anthony J. Ireland, Andy R. Ness and Jonathan R. Sandy in The Cleft Palate-Craniofacial Journal
