# Supplementary material for: Educational Attainment of Children Born with Unilateral Cleft Lip and Palate in the United Kingdom
Source: Cleft Palate Craniofac J. 2020 Sep 29;58(5):587–96. doi: 10.1177/1055665620959989 (PMC8044616; doi:10.1177/1055665620959989)
Supplement: Supplemental_material - Educational Attainment of Children Born with Unilateral Cleft Lip and Palate in the United Kingdom [file Supplemental_material.pdf]

Supplemental Table 5c:
Association between exposure variables and the proportion of pupils achieving level 2 and above in mathematics as assessed using logistic regression odds ratios, 95% confidence intervals and p values· Model 1 adjusts for month of birth gender and model 2 also adjusting for the Index of Multiple Deprivation (IMD).

Mathematics								
Variable/ Category		Max N	Model 1 (month of birth & gender)			Model 2 (month of birth, gender, IMD)		
			OR	95% CI	P	OR	95% CI	P
Dento-alveolar (vs excellent/good)	Fair	159	1·38	0·41, 4·69	0·2	1·39	0·41, 4·74	0·2
	Poor/Very poor		3·69	0·45, 30·55		3·87	0·47, 32·19	
Naso-labial appearance (vs excellent/good)	Fair	193	1·35	0·46, 3·98	0·8	1·32	0·44, 3·93	0·7
	Poor/Very poor		1·08	0·20, 5·80		1·20	0·22· 6·56	
Decayed missing filled teeth (Dmft) (vs 0)	1-3	203	0·94	0·29, 3·04	0·2	1·02	0·31, 3·35	0·3
	4+		0·49	0·17, 1·47		0·54	0·17, 1·66	
Audiology (best ear) (vs normal hearing)	Any hearing loss	164	0·67	0·22, 2·07	0·5	0·69	0·22, 2·13	0·5
Intelligibility (vs normal)	Different but intelligible	184	0·97	0·23, 4·07	0·07	0·98	0·23, 4·17	0·1
	Just intelligible or less		0·28	0·08, 1·00		0·34	0·09, 1·23	
Psychological	Low self-confidence	185	0·26	0·70, 1·00	0·05	0·29	0·08, 1·09	0·07
	Child is bullied	189	2·02	0·24, 16·66	0·5	2·28	0·26, 19·64	0·5
Minimum number of problems (vs 0-1)	2	205	1·78	0·52, 6·05	0·2	1·78	0·52, 6·06	0·3
	3-6		0·43	0·14, 1·28		0·46	0·15, 1·45	

Supplemental Table 5d:
Association between exposure variables and the proportion of pupils achieving level 2 and above in speaking and listening as assessed using logistic regression odds ratios, 95% confidence intervals and p values. Model 1 adjusts for month of birth gender and model 2 also adjusting for the Index of Multiple Deprivation (IMD).

Speaking and listening								
Variable/ Category		Max N	Model 1 (month of birth & gender)			Model 2 (month of birth, gender, IMD)		
			OR	95% CI	P	OR	95% CI	P
Dento-alveolar (vs excellent/good)	Fair	159	0·66	0·25, 1·72	0·8	0·66	0·25, 1·73	0·9
	Poor/Very poor		1·01	0·29, 3·48		1·06	0·31, 3·67	
Naso-labial appearance (vs excellent/good)	Fair	193	1·49	0·66, 3·37	0·9	1·47	0·65, 3·33	0·9
	Poor/Very poor		0·76	0·23, 2·48		0·79	0·23, 2·64	
Decayed missing filled teeth (Dmft) (vs 0)	1-3	203	0·55	0·23, 1·33	0·02	0·59	0·24, 1·44	0·06
	4+		0·35	0·14, 0·84		0·41	0·16, 1·04	
Audiology (best ear) (vs normal hearing)	Any hearing loss	164	0·52	0·21, 1·27	0·2	0·53	0·21, 1·31	0·2
Intelligibility (vs normal)	Different but intelligible	184	0·37	0·13, 1·03	<0·001	0·36	0·13, 1·02	<0·001
	Just intelligible or less		0·12	0·04, 0·33		0·13	0·04, 0·37	
Psychological	Low self-confidence	185	0·59	0·17, 2·05	0·4	0·60	0·17, 2·11	0·4
	Child is bullied	189	1·01	0·26, 3·85	>0·9	0·95	0·24, 3·81	>0·9
Minimum number of problems (vs 0-1)	2	205	0·64	0·27, 1·50	0·01	0·71	0·30, 1·70	0·02
	3-6		0·27	0·10, 0·68		0·29	0·11, 0·77	

Supplemental Table 5e:

Association between exposure variables and the proportion of pupils achieving level 2 and above in Science as assessed using logistic regression odds ratios, 95% confidence intervals and p values. Model 1 adjusts for month of birth gender and model 2 also adjusting for the Index of Multiple Deprivation (IMD).

Science								
**Variable/ Category**		**Max N**	**Model 1 (month of birth & gender)**			**Model 2 (month of birth, gender, IMD)**		
			**OR**	**95% CI**	**P**	**OR**	**95% CI**	**P**
Dento-alveolar (vs excellent/good)	Fair	159		0·22, 2·45	0·4	0·75	0·22, 2·58	0·5
	Poor/Very poor		0·58	0·15, 2·18		0·64	0·17, 2·45	
Naso-labial appearance (vs excellent/good)	Fair	193	1·29	0·47, 3·50	0·3	1·25	0·45, 3·46	0·4
	Poor/Very poor		0·39	0·11, 1·38		0·46	0·12, 1·69	
Decayed missing filled teeth (Dmft) (vs 0)	1-3	203	0·34	0·12, 0·94	0·05	0·40	0·14, 1·12	0·3
	4+		0·36	0·12, 1·09		0·55	0·17, 1·77	
Audiology (best ear) (vs normal hearing)	Any hearing loss	164	0·26	0·10, 0·70	0·008	0·25	0·09, 0·71	0·009
Intelligibility (vs normal)	Different but intelligible	184	0·77	0·21, 2·80	0·01	0·78	0·21, 2·90	0·02
	Just intelligible or less		0·18	0·06, 0·59		0·22	0·07, 0·76	
Psychological	Low self-confidence	185	0·87	0·18, 4·34	0·9	0·94	0·18, 4·82	0·9
	Child is bullied	189	0·96	0·20, 4·71	>0·9	1·07	0·20, 5·77	>0·9
Minimum number of problems (vs 0-1)	2	205	0·36	0·13, 1·03	0·003	0·41	0·14, 1·22	0·02
	3-6		0·18	0·06, 0·57		0·24	0·08, 0·77	

Supplemental Figure 2

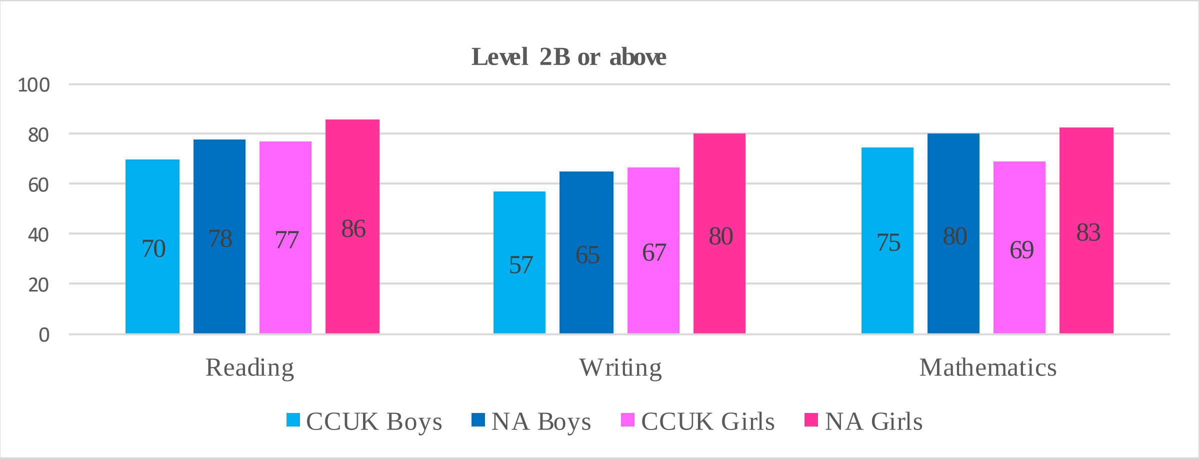

Supplemental Figure 2: Percentage of pupils scoring level 2B or above in Cleft Care UK (CCUK) vs the 2015 National Average (NA). Reading: Boys P=0·02 Girls

P=0·03, Writing: Boys P=0·05 Girls P= 0·01, Mathematics: Boys P= 0·1 Girls P=0·002
